# Recent Advances in *Momordica charantia*: Functional Components and Biological Activities

**DOI:** 10.3390/ijms18122555

**Published:** 2017-11-28

**Authors:** Shuo Jia, Mingyue Shen, Fan Zhang, Jianhua Xie

**Affiliations:** State Key Laboratory of Food Science and Technology, Nanchang University, Nanchang 330047, China; jiashuo@email.ncu.edu.cn (S.J.); shenmingyue1107@ncu.edu.cn (M.S.); zhangfan@email.ncu.edu.cn (F.Z.)

**Keywords:** chemical components, *Momordica charantia*, phytochemistry, biological activities

## Abstract

*Momordica charantia* L. (*M. charantia*), a member of the *Cucurbitaceae* family, is widely distributed in tropical and subtropical regions of the world. It has been used in folk medicine for the treatment of diabetes mellitus, and its fruit has been used as a vegetable for thousands of years. Phytochemicals including proteins, polysaccharides, flavonoids, triterpenes, saponins, ascorbic acid and steroids have been found in this plant. Various biological activities of *M. charantia* have been reported, such as antihyperglycemic, antibacterial, antiviral, antitumor, immunomodulation, antioxidant, antidiabetic, anthelmintic, antimutagenic, antiulcer, antilipolytic, antifertility, hepatoprotective, anticancer and anti-inflammatory activities. However, both in vitro and in vivo studies have also demonstrated that *M. charantia* may also exert toxic or adverse effects under different conditions. This review addresses the chemical constituents of *M. charantia* and discusses their pharmacological activities as well as their adverse effects, aimed at providing a comprehensive overview of the phytochemistry and biological activities of *M. charantia*.

## 1. Introduction

**Figure 1:**
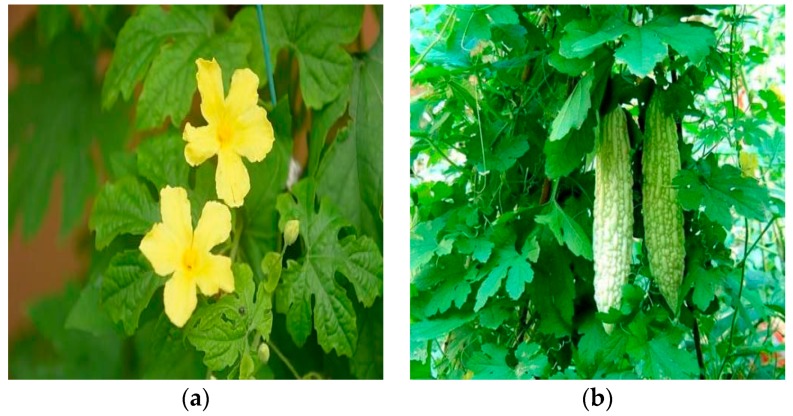
The above two pictures show the morphological characteristics of the *M. charantia*: (**a**) leaf and flowers (**b**) unripe fruits.

Since ancient times, a variety of plants have been used as medicine and vegetable throughout the world. The combination of medicine and vegetable usage have made *Momordica charantia* (*Momordica* species) popular for thousands of years. *Momordica charantia* (*M. charantia*) (Figure 1), a valuable plant, belongs to the *Cucurbitaceae* family; it is commonly known as bitter gourd, balsam pear, bitter melon, kugua or karela [1]. The generic name “*Momordica*” comes from Latin, meaning “to bite”, which refers to its leaf with serrated edges which looks as if it has been bitten [2]. The plant lives up to its common name “bitter melon” or “bitter gourd”, as all parts of the plant including the fruit taste very bitter [2,3]. *M. charantia* is widely cultivated in the tropical and subtropical regions of the world, such as India, Malaya, China, Thailand, Japan, Singapore, Vietnam, Amazon, East Africa, Brazil, China, Colombia, Cuba, Ghana, Haiti, India, Mexico, Malaya, New Zealand, Nicaragua, Panama, Middle East, Central and South America [4,5,6]. The fruit of *M. charantia* is oblong or spindle with pimples on the surface and resembles a small cucumber; young fruit is emerald green that turns orange when ripe [6,7], while the flesh becomes scarlet from white during maturation. The fruit can be used as food material in every stage between maturing, and it is commonly used as a vegetable in different parts of the world.

**Figure 2:**
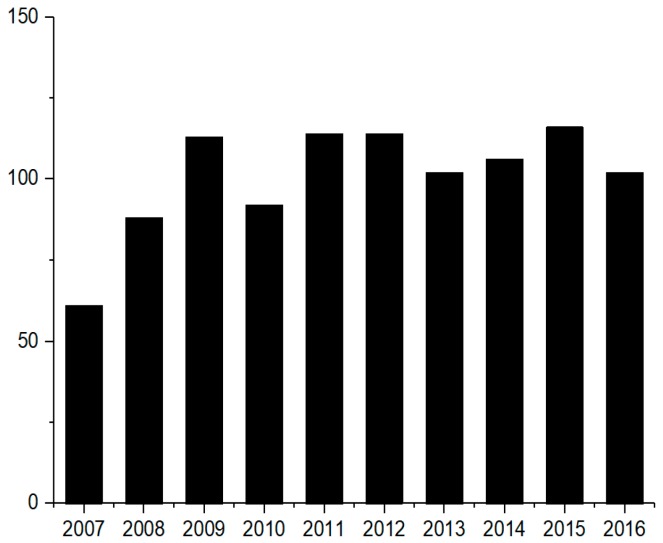
Articles published in Web of Science with *M. charantia* as a keyword in the last decade.

Although its fruit has a special bitter taste, *M. charantia* is popular among different people in the world. The phenomenon may be explained not only by its special taste but also the various bioactive effects which general vegetables do not provide. In many countries and regions, *M. charantia* also has been used as herbal medicine. The whole plant, especially the seeds and fruit, have significant pharmacological effects; for example, it has been used in the treatment of diabetes since ancient times, and still plays an important role in the prevention and remedy of diabetes in many developing countries [8,9]. Fractions of *M. charantia,* such as fruits, vines, leaves and even roots have been used as folk medicine for the remedy of diseases like toothache, diarrhea, furuncle and diabetes. Relevant products of *M. charantia* are quite popular now; for example the bitter gourd tea, which is known as gohyah or herbal tea made from dried slices, is applied mainly for medicinal purposes. The number of related articles published each year on the SCI website (Figure 2) demonstrate the steady and increasing trend in the number of research papers produced per year with *M. charantia* as a keyword. This plant is a traditional herbal medicine, possesses various pharmacological functions, namely antidiabetic, abortifacient, anthelmintic, contraceptive, antimalarial and laxative. It is used for the treatment of dysmenorrhea, eczema, gout, jaundice, leprosy, piles, pneumonia, psoriasis, rheumatism and scabies [8,10,11].

Several medicinal properties of *M. charantia* have been studied that include hypoglycemic, anti-bacterial, anti-viral, anti-tumor, immunomodulation, anti-oxidant, anti-diabetes, anthelmintic, antimutagenic, antilipolytic, antifertility, hepatoprotective and anti-inflammatory activities, as well as anti-ulcerogenic, anti-oxidative and immune-modulatory acivities. [12,13,14]. In vitro, studies have confirmed that *M. charantia* proteins (α- and β-momorcharin) have inhibitory effect against human immune deficiency virus (HIV). Its extract can also be used as a broad-spectrum antibacterial agent to fight off infections [15]. These beneficial effects are attributed to the various bioactive components of *M. charantia*, which are important sources of phytoconstituents used to treat various diseases since ancient times [16,17].

Though *M. charantia* possesses various pharmacological activities, there are also adverse effects that have been reported in the past years which limit its wider application. In addition to some toxic symptoms, previous studies have concluded that *M. charantia* may induce symptoms such as hypoglycemic coma in children, abortion or even death in laboratory animals [6].

This paper reviews various aspects of the results of investigations involving *M. charantia* in the recent years. It is aimed at providing a comprehensive overview of the phytochemistry and commercial application aspects of *M. charantia* to attract more attention to their biological activities, and to inform people for better utilization of *M. charantia*.

## 2. Chemical Composition

Several bioactive compounds of *M. charantia* fruit have been recorded in the literature; they are classified as carbohydrates, proteins, lipids and more [18,19,20]. *M. charantia* contains triterpenoids [21,22,23,24], saponins [25,26,27], polypeptides [28], flavonoids [29], alkaloids [30] and sterols [23]. Previous phytochemical studies have shown the bioactive components and their related functions (Table 1).

### 2.1. Polysaccharides

Polysaccharides are among the important bioactive components of *M. charantia*. It has been shown that polysaccharides from *M. charantia* fruits possess various bioactivities, such as antioxidant, antidiabetic, immune enhancing, neuroprotective, antitumor and antimicrobial [31,32,33,34,35,36,63].

Extraction methods [64,65,66] such as traditional hot-water, acid and alkali extractions, as well as microwave-, ultrasonic- and enzymatic-assisted extractions, followed by ethanol precipitation have been applied for the separation of crude polysaccharides from *M. charantia*. Polysaccharides make up approximately 6% of bitter gourd powder, are classified as heteropolysaccharide, and are composed of galactose (Gal), glucose (Glu), arabinose (Ara), rhamnose (Rha) and mannose (Man) [66]. Deng et al. [67] showed that the contents of polysaccharides may be influenced by different conditions; the polysaccharide contents in 13 cultivated varieties range from 5.91% to 10.62% of dry powder. In addition, polysaccharides are classified in two main fractions; one of them has an average molecular weight (*Mw*) in the range of 1558.88–3048.56 kDa, and the other group is in the 33.1–58.74 kDa range. Tan and Gan [68] reported that an acidic and branched heteropolysaccharide (MCBP) isolated from *M. charantia* with a *Mw* of 92 kDa was mainly composed of Man, galacturonic acid (GalA), Rha, Glu, Gal, xylose (Xyl) and Ara with molar ratios of 0.01:0.15:0.02:0.38:0.31:0.05:0.09, respectively. Furthermore, MCBP possessed antioxidant, α-amylase inhibition and angiotensin-converting enzyme inhibition functions. A pectic polysaccharide (PS) was isolated and identified from *M. charantia*; its backbone is mainly composed of [4)-α-d-Gal*p*A6Me-(1]_3_→4)-α-d-GalpA6Me-(1→ [63]. It was composed of 1,4,5-tri-*O*-acetyl-2,3,6-tri-*O*-methyl-d-galactitol, 1,5-di-*O*-acetyl-2,3,4,6-tetra-*O*-methyl-d-galactitol, and 1,2,4,5-tetra-*O*-acetyl-3,6-di-*O*-methyl-d-galactitol, in the ratio of 3:1:1 and with *Mw* of 2 × 10^4^ Da [63]. Recently, a water-soluble polysaccharide (MBP) was isolated from *M. charantia* fruits, and mainly composed of Ara, Xyl, Gal and Rha in a molar ratio of 1.00:1.12:4.07:1.79, with *Mw* of 1.15 × 10^6^ Da; it showed a significant hypoglycemic effect [31]. In particular, Raish [69] demonstrated that *M. charantia* polysaccharides ameliorate oxidative stress, hyperlipidemia, inflammation and apoptosis during myocardial infarction by inhibiting the NF-κB signaling pathway. *M. charantia* polysaccharides also had the ability to enhance total volatile fatty acids production, modulate the rumen fermentation pathway and influence the number of cellulolytic bacteria population [70].

### 2.2. Proteins and Peptides

Proteins and peptides are also the main functional components in the fruit and seeds of *M. charantia*. Many types of proteins and peptides have been isolated from different parts of *M. charantia*, such as ribosome inactivating proteins (RIPs), *Momordica charantia* lectin (MCL), Momordica anti-HIV protein of 30 kD (MAP30), α-momorcharin (α-MMC), β-momorcharin (β-MMC), γ-momorcharin, δ-momorcharin and ε-momorcharin, which possess RNA *N*-glycosidase activity, PAG activity, DNase-like activity, phospholipase activity, superoxide dismutase activity, anti-tumour, anticancer, immunosuppressive and anti-microbial activity [37,38,39,40,41,42,43].

RIPs are a kind of RNA glycosylases that cleave an adenine–ribose glycosidic bond; it is a type of alkaline protein, which can inhibit the process of protein synthesis by inactivating ribosomes. They can be further divided into three classes; RIPs with only a RIP chain are classified as type I, and the structure of type II RIPs generally has two chains, A and B, which are interconnected by disulfide bonds [71]. And the structure of B chain allow them binding with galactose residues on the oligosaccharide chain. There are also atypical type I RIPs (on the basis of their structure) which are classified as type III RIP [72].

*M. charantia* lectin (Type II RIP) and α-MMC have been isolated from *M. charantia* seeds; it can significantly inhibit human nasopharyngeal cancer cells and xenograft tumors in vitro [43]. MCL is a type II RIP, known to be particularly toxic, and has been used as an anti-tumor agent [73]. Momordicin is also a type II (single-stranded) RIP that has been successfully isolated from *M. charantia* together with other factors.

MAP30 is a single chain RIP, named for its molecular mass of 30 kD; it has been found to have strong anti-tumor potential similar to MCL [38,71]. The protein also significantly inhibits proliferation and causes apoptosis in a panel of cancer cells from prostate, breast, lung, hepatocellular and brain glioblastoma [38]. The MAP30 protein consists of 286 amino acids and the mature protein contains one *N*-glycosylation site and a glycosylase that aids in the binding of elongation factors [38].

Like MAP30, both α-MMC and β-MMC are type I RIPs, containing only one enzymatic chain [72]. α-MMC is also a 30-kDa glycoprotein, while β-MMC is slightly smaller (29-kD) glycoprotein. Both have anti-tumor activity individually.

Polypeptide-P, a hypoglycemic peptide, is a kind of carbohydrate binding protein secreted by plant cells; it plays an important role in cell recognition and adhesion reactions. It is isolated from the fruit, seeds and tissues of *M. charantia* with a *Mw* of approximately 11 kD; it contains 166 amino acid residues and another polypeptide with a *Mw* of 3.4 kD has also been isolated from bitter melon [74]. Other proteins and peptides, such as peroxidase (43 kDa), *Momordica* cyclic peptides [75], trypsin inhibitors (McTI-I, -II and -III), cystine knot peptides, RNase MC2 (14 kDa), antifungal protein and MCha-Pr have also been isolated from *M. charantia* [76].

### 2.3. Saponins and Terpenoids

Saponins are a class of glycosides in which the aglycone is a triterpenoid or a spiro-steroid compound. All of the compositions are of sugar and aglycone, and the difference between them lies in the structure of aglycones. Saponins are found in the roots, stems, leaves and fruit of the *M. charantia*. Research has shown that the major chemical constituents are tetracyclic triterpenoids and their glycosides, most of which are referred to as cucurbitanes, and are well-known for their bitterness and toxicity. The content of total saponins in *M. charantia* powder is about 0.0432% [77].

The saponins substances are the active ingredients of multiple drugs, widely distributed in a variety of plants [78], which contain triterpenoidal saponins (e.g., cucurbitacin alkyl type, oleanane type, ursane type) and steroidal saponins. The cucurbitacins are a group of bitter-tasting, highly-oxygenated, mainly tetracyclic, triterpenic plant substances derived from the cucurbitane skeleton. Many pharmacological studies further indicated that cucurbitanes from *M. charantia* are responsible for their anti-diabetic and hypoglycaemia activities [79]. Cucurbitane-type compounds, such as goyaglycosides a, b, c, d, e, f, g and h; goyasaponins I, II, and III; and momordicosides A, C, F_1_, I and K have been isolated from the methanolic extract of *M. charantia* fruits [27]. Cucurbitane-type triterpenoids: β,19-epoxy-3β,25-dihydroxycucurbita-6,23(*E*)-diene, and 3β,7β,25-trihydroxycucurbita-5,23(*E*)-dien-19-al were isolated from the methanol extract of *M. charantia* dried gourds, which could lower blood sugar in diabetic mice. Moreover, Harinantenaina et al. [80] also demonstrated that compounds of *M. charantia* have hypoglycaemic effects in vivo. Chang et al. [55] isolated four new cucurbitane-type triterpenes, cucurbita-5,23(*E*)-diene-3β,7β,25-triol, 3β-acetoxy-7β-methoxycucurbita-5,23(*E*)-dien-25-ol, cucurbita-5(10),6,23(*E*)-triene-3β,25-diol and cucurbita-5,24-diene-3,7,23-trione, from the methyl alcohol extract of *M. charantia* stems. In 2011, five kinds of saponins and cucurbitane triterpenoids, including 3β,7β,25-trihydroxycucurbita-5,23(*E*)-dien-19-al, momordicine I, momordicine II, 3-hydroxycucurbita-5,24-dien-19-al-7,23-di-*O*-β-glucopyranoside and kuguaglycoside G were isolated from *M. charantia.* In another study, eight new cucurbitane-type glycosides, kugua saponins A–H and six known compounds, were isolated by the directed fractionation of *M. charantia* fruits [81]. Zhang et al. [82] also reported that four new cucurbitane-type triterpenes, (23*R*)-7β-hydroxy-3β-*O*-malonyl-23-methoxycucurbita-5,24-diene-19-al, (23*E*)-7β,25-dihydroxy-3β-*O*-methylmalonylcucurbita-5,23-diene-19-al, (23*E*)-7β-hydroxy-3β-*O*-methylmalonyl-25-methoxycucurbita-5,23-diene-19-al, (23*E*)-7β,25-dihydroxy-3β-*O*-crotonylcucurbita-5,23-diene-19-al, and one new glycoside 7β-hydroxy-3β-*O*-malonyl-cucurbita-5,24-diene-19-a-23-*O*-β-d-glucopyranoside, were isolated from the rattans of wild *M. charantia*.

### 2.4. Flavonoids and Phenolic Compounds

Flavonoids and phenolic compounds are important components of *M. charantia* [5,83]. They include gallic acid, protocatechuic acid, gentistic acid, (+)-catechin, vanillic acid, syringic acid, (−)-epicatchin, p-coumaric acid, benzoic acid, sinapinic acid, o-coumaric acid, chlorogenic acid, t-cinnamic acid and t-ferulic acid. The most abundant flavonoids, quinic acid (145.279 ng/mg) and catechin (57.24 ng/mg), were determined in the BME4 <3.5 kDa (methanol hydrophilic extraction of *M. charantia* dialysis tubing with 3.5 kDa) by UPLC-MS [84]. Phenolic acid constituents were distributed in various amounts for each phenolic acid among a variety or parts of tissues [85]. In *M. charantia* flesh, the main phenolic acids were gallic acid, gentisic acid, catechin, chlorogenic acid and epicatechin, and ranged from 8.04 to 39.76, 16.99 to 32.39, 23.06 to 82.45, 4.55 to 15.83, and 16.14 to 44.28 mg/100 g dry material [85]. Ethyl acetate crude extract of *M. charantia* contained ascorbic acid (576.5 ng/mg), 3-coumaric acid (528.55 ng/mg), luteolin-7-*O*-glycoside (725.50 ng/mg), apigenin-7-*O*-glycoside (1955.55 ng/mg), caffeic acid (215.6 ng/mg) and naringenin-7-*O*-glycoside (181.30 ng/mg) [84]. The amounts of protocatechuic acid, p-coumaric acid, syringic acid, vanillic acid and benzoic acid ranged from 2.07 to 8.78, 1.83 to 8.23, 1.77 to 3.67 and trace to 2.42 mg/100 g dry material in the flesh of all varieties of the bitter melons, respectively [85].

Catechin and epicatechin are the two most common flavonoids in plants. Budrat and Shotipruk [86] revealed that catechin is the highest phenolic acid contained in bitter melon (46.16 mg/g dry weight, 72–86% of the total phenolic contents) from the extracts obtained by subcritical water extraction, followed by gentisic acid (4–12%), gallic acid (0.25–0.87%) and chlorogenic acid (0–0.26%), respectively. Main phenolic constituents in the extracts were catechin, gallic acid, gentisic acid, chlorogenic acid and epicatechin [87]. Caffeic acid is classified as a phenylpropanoid; the concentration of caffeic acid in bitter melon was found to be 3.55 mg/L in the methanolic fraction [88]. p-coumaric acid, tannic acid, benzoic acid, ferulic acid, gallic acid, caffeic acid, and (+)-catechin have also been found in aqueous extract fractions of *M. charantia*.

### 2.5. Other Components

Besides bioactive ingredients, unsaturated fatty acids, alkaloids, amino acids and minerals, vitamins are also contained in *M. charantia* [8,89,90,91,92]. The proportion of unsaturated fatty acid component in bitter melon is relatively high; monounsaturated fatty acids in the ratio of total fatty acid content are about 20.1%, while polyunsaturated fatty acid content is about 64.3%. Nine kinds of unsaturated fatty acids have been found in bitter melon extracts [84]. It has also been demonstrated that 12, 13 and 12 fatty acids are found in young, mature, and senescent leaves of *M. charantia* L., representing 87.3%, 95.25%, and 83.11% of the total fatty acids [93]. The contents of total amino acids and the free amino acids of *M. charantia* were 11.99% and 2.36% as determined by acid hydrolysis and amino acid analysis [94]. In addition, bitter melon is a natural source of vitamins; ascorbic acid was detected in the range of 440–780 mg in the fruit fraction [95].

## 3. Biological Activities

As *M. charantia* has been used for the treatment of various kinds of diseases since ancient times, it is still widely applied for therapy in Latin America and Asian countries as mentioned above. The following is an overview of its common pharmacological activities.

### 3.1. Antidiabetic Activity

Diabetes mellitus, one of the fastest growing diseases in the world, is a group of metabolic diseases characterized by hyperglycemia resulting from defects in insulin secretion, insulin action, or both [96]. Many studies suggest that a variety of *M. charantia* extract can be used as a remedy for the treatment of diabetes [8,31,97,98,99,100,101]. It has also been widely used as an antidiabetic drug in different countries for thousands of years [8,10,102].

Many studies have demonstrated that *M. charantia* has potent antidiabetic activities through cell-based assays, animal models and human clinical trials [6,103,104,105,106]. Oral administration of the aqueous extract *M. charantia* fruits could significantly lower blood glucose level in streptozotocin- (STZ-) induced diabetic rats at a dose of 250 mg/kg [107]. The aqueous extract of *M. charantia* fruits can stimulate insulin secretion of β cells in pancreatic islets isolated from obese-hyperglycemic mice [102]. Another study showed that *M. charantia* fruit aqueous extract also has hypoglycaemic activity in cyproheptadine-induced diabetic mice [108]. Orally administered *M. charantia* aqueous extracts lowered glucose concentrations independently of intestinal glucose absorption and involved extrapancreatic effects [103]. It also plays a role in the renewal of β cells in STZ-diabetic rats or recovery of destroyed β cells [12]. A boiling water extract from *M. charantia* has significant repairing effects on HIT-T15 cells against superoxide anion radicals, which showed potential cell repairing activity on alloxan-damaged HIT-T15 pancreatic β cells; its fraction with a *Mw* below 3 kDa (2%) performed better in stimulating insulin secretion [109]. It has also been reported that in clinical trials, polypeptide-P isolated from the bitter gourd was found to have hypoglycemic activity [110]. With the chronic administration of the *M. charantia* fruit juice at 20 mg/kg orally, blood glucose tolerance of alloxan-induced rats was ameliorated significantly from day 7 to day 22, and was reduced to normal levels [111]. The major pure cucurbutanoid compounds of *M. charantia*, 5β,19-epoxy-3β,25-dihydroxycucurbita-6,23(*E*)-diene, and 3β,7β,25-trihydroxycucurbita-5,23(*E*)-dien-19-al have been demonstrated to have hypoglycaemic effects in the diabetes-induced male ddY mice. Although the glucose lowering effects are lower than glibenclamide at the same concentration (400 mg/kg), they are still significant [80]. *M. charantia* fruit juice could significantly reduce blood glucose levels in alloxan-induced diabetic rats, and could restore the impaired estrous cycle in diabetic rats. The mechanisms by which *M. charantia* extracts act on diabetes are via both intra- and extra-pancreatic mechanisms [112]. Fernandes et al. [113] suggested that the antidiabetic mechanism of *M. charantia* extracts may be due to enhancing insulin secretion by the islets of Langerhans, reducing glycogenesis in liver tissue, enhancing peripheral glucose utilization and increasing serum protein levels.

### 3.2. Anti-Oxidant Activity

Many studies have demonstrated that *M. charantia* is a good natural source of antioxidants under experimental conditions; it possess an activity against oxidant damage in vitro and in vivo [48,60,114]; the bioactive phytochemicals mainly include polysaccharides, saponins and phenolics [9,91]. Bitter gourd pulp and its extracts, followed by seed powder and its ethanol/water extracts exhibited stronger anti-oxygenic activity than other solvent extracts, which were determined via several in vitro models [115]. ABTS radical cation-scavenging assays demonstrated that three new triterpenoids compounds isolated from *M. charantia* stems had a weaker effect compared to the control group, IC_50_ values were 268.5 ± 7.9, 352.1 ± 11.5 and 458.9 ± 13.0 µM, respectively. But cucurbita-1(10),5,22, 24-tetraen-3a-ol showed a significant inhibition on XO at 100 µM [48]. Supplementation of *M. charantia* (13.33 g/kg) in diabetic rats significantly decreases (*p <* 0.001) TBARS levels and significantly increases antioxidants (SOD, CAT and GST) activities [116]. Oral administration of *M. charantia* lyophilized powder plays an important role in decreasing serum TBARS and maintaining the GSH content in alloxan-induced diabetes rats [104]. It has been reported that a wild variety of bitter gourd alcohol extract and aqueous extract have an effect in eliminating 1,1-diphenyl-2-trinitrobenzene hydrazine (DPPH) radical at 300 µg/mL, as well as metal chelating activity at 100, 250, 500 µg/mL when compared with VE, but less capacity on inhibiting peroxidase and lipid peroxidation at all concentrations in different tissues [117]. The aqueous extract reduced the serum AST, ALT and NO content and the expression of hepatic iNOS; it protected liver from the damage-induced by mitochondrial ROS at each dose (250, 500 and 750 mg/kg) in the experiment [118]. After continuous administration for eight weeks (thrice a week, 300 mg/kg), *M. charantia* alcoholic extract exerted antioxidant potentials by reversing the oxidant/antioxidant imbalance; changes in liver markers also suggest the extract maintained cellular integrity of the liver tissue in ammonium chloride-(AC-) induced hyperammonemia rats [114].

The antioxidant activities of the aqueous extracts of *M. charantia* pulp were evaluated using assays to assess DPPH and hydroxyl radical scavenging activities, metal-chelating activity and reducing power of the extracts [119]. The activity of a water-soluble pectic polysaccharide isolated from the hot water extract of the unripe *M. charantia* fruits on free radicals scavenging ability was assessed to be EC_50_ = 2.22 mg/mL [64].

Flavonoids are known to be one of the most effective free radical scavengers and antioxidants from *M. charantia*. The antioxidant capacity enhanced gradually with the increase of flavonoid concentration, and the scavenging efficiency even reached 96.14 ± 1.02% at the concentration of 1.2 mg/mL [5]. While there was also a big difference on antioxidant capacity between them, among the 13 substances identified as cucurbitane-type triterpene glycosides, compound **1** showed weak DPPH-scavenging activity but strong inhibitory effect on XO and ABTS radical. Antioxidant capacity was expressed as O_2_- scavenging activity for other compounds such as compound **2**–**4**. Oxygen radical absorbance capacity (ORAC)-pyrogallol red (PGR) values were also differed between compounds **2** and **3** (0.88 ± 0.02 and 0.55 ± 0.09, respectively) [120]. 9c, 11t, 13t-conjugated linolic acid (CLN), mainly distributed in the bitter gourd seed, could significantly increase acyl CoA oxidase activity in a peroxisome proliferator responsive murine hepatoma cell line, H4IIEC3, and was identified as a PPARα activator in wild bitter gourd, which could act on PPARα signaling pathways [92]. For H_2_O_2_ and HX-XO-induced oxidative damage models, total phenolic extracts from *M. charantia* showed a dose-dependent antioxidant effect on NIH 3T3 cardiac fibroblasts and A431 keratinocytes at the range of 50–300 µg/mL; the extract protected both cell lines from the damage of H_2_O_2_ at a concentration of 1 × 10^−4^ mol/L [121].

### 3.3. Antiviral Activity

Ethanolic extracts from leaves and stems of *M. charantia* highly inhibit HSV-1 and SINV viruses, and research also suggests that the antiviral activity reflects a close dependence on photosensitizer(s) rather than momordicin I or II [122]. A variety of compounds isolated from *M. charantia* have antiviral activity; many of them are proteins and steroids [123,124]. Kuguacin C and Kuguacin E isolated from the root of *M. charantia* showed moderate anti-HIV-1 activity with EC_50_ values of 8.45 and 25.62 µg/mL, while exerting minimal cytotoxicity on uninfected C8166 cells (IC_50_ > 200 µg/mL) [21]. MAP30 is the main component of antiviral activity in vitro; it selectively kills lymphocytes and macrophage infected by HIV, inhibits HIV-I virus DNA replication in monocytes, while exerting minimal cytotoxicity on uninfected cells [125]. Similarly, research also found that MAP30 of bitter gourd proteins can inhibit HIV activity, depress the expression of the virus core protein p24 and viral-associated reverse transcriptase (HIV-RT), while having less effect on cellular DNA or protein synthesis in H9 cells [126]. MRK29, as a lectin isolated from *M. charantia*, was found to act through inhibition of viral reverse transcriptase [127]. Momordicin had direct protective effect on Coxsackie virus (CVB3)-infected myocardiocyte, and depressed RNA transcription and translation of CVB3 in myocardial cells [128].

### 3.4. Antimicrobial Activity

Essential oils of *M. charantia* seeds have significant inhibitory effect on *S. aureus*, while having less impact on *E. coli* and *C. albicans* [129]. The aqueous extract from *M. charantia* seed exhibited significant antimicrobial activity against several bacteria in the following ascending order: *P. multocida*, *S. typhi*, *S. epidermidis* and *L. bulgaricus*. As for the ethanolic extract, the sequence was *S. aureus*, *M. luteus*, *E. coli*, *S. epidermidis* and *L. bulgaricus*, while n-hexane and petroleum ether extracts were effective against *S. aureus* [130].

*M. charantia* pulp extract has been proven to have broad-spectrum antimicrobial activity [131], the same as the hydrophilic leaf extracts, which exhibited antibacterial activities against *E. coli*, *Staphylococcus*, *Pseudomonas*, *Salmonella* and *Streptobacillus*. This may be attributed to 5-a-stigmasta-7, 25-dien-3-b-ol, elasterol and lanosterol [15]. Ethanol extracts of *M. charantia* leaves exhibit inhibition on *B. cereus* and *S. aureus.* The ethanol fraction has no apparent effect on *E. coli*, which is in contrast with the treatment of the ethyl acetate extracts [132]. Methanolic extract from *M. charantia* leaves showed the strongest antibacterial activity amongst several organic solvent extracts, with a significant inhibitory effect on *E. coli* and *S. aureus* [133].

No inhibitory activity was observed against methicillin-resistant *S. aureus* or *P. aeruginosa* in either the hydrophilic or methanolic extracts of several wild *M. charantia* L. var. abbreviata Seringe cultivals, but some of them showed strong inhibitory effects on the growth of *E. coli* and *S. enterica* [134]. A low molecular mass peptide (approximately 10 kDa) purified from *M. charantia* is more effective against *S. aureus* and *E. coli* as compared to *S. typhi* and *P. aeruginosa.* As the concentration increased to 200 µg/mL, apparent proliferation ratios of *S. aureus*, *E. coli*, *S. typhi* and *P. aeruginosa* were decreased by 57%, 49%, 29% and 18%, respectively [42]. α-MMC isolated from *M. charantia* strongly inhibited *P. aeruginosa* and the mycelial growth of *F. solani* and *F. oxysporum* [135]. The seed extracts also significantly inhibited the growth of *F. solani* in a dose-dependent manner, the probable explanation is that it undermined the integrity of the cell nucleus and DNA [136].

### 3.5. Anti-Inflammatory Activity

Oral administration of 2% and 5% *M. charantia* dry powder significantly depressed macrophage infiltration in epididymal adipose tissues (EAT) and brown adipose tissues (BAT) of rats fed with high-fat diet (HFD), and downregulated the expression of pro-inflammatory cytokine monocyte chemotactic protein-1, TNF-α and IL-6 in EAT [137]. However, an opposite result was observed which is that bitter melon powder could significantly improve the pro-inflammatory cytokines (TNF-α, IL-6) and anti-inflammatory cytokine (IL-10) via suppressing the activation of NF-κB signaling pathways [138]. *M. charantia* normalized the content of neuroinflammatory markers (e.g., NF-B1, TNF-α, IL-16, IL-22, IL-17R), significantly reduced brain oxidative stress induced by high fat diet administration and effectively prevented neuroinflammation [139]. Studies also found *M. charantia* suppressed the secretion of IL-7 and promoted the secretions of TGF-β and IL-10, thereby leading to the decrease of lymphocytes and elevation in Th cells and natural killer (NK) cells in vivo [140].

Research on a cerebral ischemia-reperfusion injury model in male Sprague Dawley rats shows that *M. charantia* polysaccharides have neuroprotective effects against global cerebral ischemia/reperfusion injury by scavenging radicals (O_2_-, NO and ONOO-) and reduces neural cell death in vitro; it also inhibits the release of cytochrome C, phosphorylation of JNK3 and expression of Fas-L in both pre-ischemia and post-ischemia treatment [141]. Wild *M. charantia* in diets attenuated inflammatory stress in mice with sepsis through reduced secretions of pro-inflammatory cytokines and the expression of proteins (COX-2, iNOS and NF-κB) associated with inflammation [142]. *M. charantia* polysaccharides enhanced the activity and production of superoxide dismutase, catalase, non-protein sulfhydryls and Bcl-2 in pretreated rats prior to isoproterenol-induced myocardial infarction, along with the expression of proinflammatory cytokines (IL-6 and IL-10), while inflammatory markers (nitric oxide, myeloperoxidase, and inducible NO synthase) and apoptotic markers (caspase-3 and BAX) were down-regulated [71]. For cyclophosphamide-treated mice, *M. charantia* polysaccharides normalized immunological parameters and there was no significant difference between the high-dose group (300 mg/kg/day) and normal control at day 30. The immunomodulatory activity mainly showed improved phagocytosis and NK cell vitality in comparison with the model control [33].

Total phenolic extracts of *M. charantia* significantly attenuated *P. acnes*-induced inflammatory responses, inhibited infiltrations of neutrophils and IL-1β leukocytes and NF-κB activation, depressed MMP-9 levels and the production of IL-8, IL-1β, TNF-α in vitro, and inactivated mitogen-activated protein kinase (MAPK) [143].

### 3.6. Anti-Tumor Activity

*M. charantia* extracts and its monomer components have shown strong anticancer activity against various tumors such as lymphoid leukemia, lymphoma, choriocarcinoma, melanoma, breast cancer, skin cancer and prostate cancer [6]. Anti-CD5 monoclonal antibodies linked to momordin (a ribosome-inactivating protein purified from *M. charantia*) performed better than other anti-CD5-based immunoconjugates containing ricin A chain on human T cell leukemia Jurkat. In the model of nu/nu mice bearing Jurkat leukemia, animals treated with the immunotoxin suffered smaller tumor size and significant inhibition (*p <*0.01) of the tumor development was observed at day 120 [144].

Whole fruit extracts of *M. charantia* elevated hepatic GST and –SH levels, significantly reduced the tumor burden in DMBA-induced papillomagenesis, and in the groups, no skin papillomas were observed during the entire experimental period [145]. As for PN-induced and TPA-promoted papillomagenesis in mice, the formation of papillomas was delayed and the mean numbers of papillomas per mouse were approximately reduced by 33% and 36% in two groups treated with the triterpenes [25]. *M. charantia* seeds also exhibited strong inhibitory activity on tumor cells in vivo [146]. Research has demonstarted the effect on Su9T01, HUT-102 and Jurkat cells when compared with extracts of other plants, and the inhibitory effect on cell proliferation may partly be attributed to α-eleostearic acid [147]. α-eleostearic acid, which is known as the major component in *M. charantia* seeds, as well as its dihydroxy derivative, has been proved to be the most effective antitumor agent extracted by ethanol; it strongly inhibited the growth of some cancer and fibroblast cell lines, including those of HL60 leukemia and HT29 colon carcinoma [148]. Eleostearic acid inhibited the proliferation of both breast cancer cell lines of estrogen receptor (ER) α-negative and ER α-positive and induced G2-M block in the cell cycle and apoptosis [149].

Matrix metalloproteinases (MMPs) play an important role in the degradation of the extracellular matrix and are closely associated with the occurrence and promotion of many diseases, such as tumor invasion, metastasis and neovascularization in pathological cases. Therefore, blocking the degradation of extracellular matrix and inhibiting the activity of MMPs has gradually become a new target for tumor therapy [150]. Studies found that ethanol extracts of bitter gourd leaves can significantly reduce the transfer and invasion of prostate cells in vitro by depressing the secretion of MMP-2 and MMP-9 [151]. Its methanolic extracts inhibited the motility of human lung adenocarcinoma CL1 series of cell lines in a dose-dependent manner and depressed the activity of enzymes related to metastases. For CL1-0 and CL1-5, methanolic extract inhibited Src and FAK to varying degrees, which play an important role in the process of tumor invasion to malignant invasion phenotype [152].

*M. charantia* juice activated AMPKs in human pancreatic carcinoma cells, decreased cell viability in all four pancreatic carcinoma cell lines (BxPC-3, MiaPaCa-2, AsPC-1 and Capan-2 cells), exerted strong apoptosis-inducing activity and significantly inhibited MiaPaCa-2 tumor xenograft growth without noticeable toxicity in nude mice [153]. Prostate cancer cells (human prostate cancer cells, PC3 and LNCaP) treated with *M. charantia* extracts accumulated S phase cell populations, modulated cyclin D1, cyclin E, and p21 expression, enhanced Bax expression, induced PARP cleavage and delayed the progression to high-grade prostatic intraepithelial neoplasia in TRAMP (transgenic adenocarcinoma of mouse prostate) mice [154]. The inhibition effect was not due to a cytotoxic effects of the *M. charantia* aqueous extract but rather to blocking the growth of prostate adenocarcinoma cells and decreasing the basal level of cyclic GMP in vitro and in vivo [155]. MAP30 recombinant protein, which was expressed by *E. coli* BL21 (DE3) cells, inhibited the growth of bladder cancer 5637 cells by inducing apoptosis in a dose- and time-dependent manner at 100, 200 and 400 µg/mL [156]. RNase MC2 isolated from *M. charantia* has been found to inhibit the proliferation of Hep G2, lead to cell cycle arrest and apoptosis [157]. Treatment of MCF-7 cells with RNase MC2 caused nuclear damage and finally resulted in early/late apoptosis, and early apoptosis was induced in a dose-escalating manner after exposure to increasing concentrations of RNase MC2 [158].

GADD45 was identified to be a critical mediator of apoptosis triggered by the activation of JNK and/or p38, via MTK1/MEKK4 MAPK signaling pathways [159]. *M. charantia* seed oil treatment upregulated GADD45, p53 and PPARγ mRNA expression, and thereby induced apoptosis in Caco-2 cells, and acted better than troglitazone at 25 µM [160]. As for the azoxymethane (AOM)-induced colonic aberrant crypt foci model, dietary supplementation with seed oil from *M. charantia* enhanced expression of PPAR protein levels and significantly reduce the incidence and the multiplicity of tumors [161]. Although the mechanism of how AMPK activation plays a role in tumor cells has not been fully understood yet, Kwatra et al. [162] found that methanol extract of *M. charantia* fruit can activate AMPK by reducing intracellular ATP levels, leading to tumor cell autophagy of colon cancer stem cells and ancestral cells.

P-glycoprotein (P-gp) is a transmembrane glycoprotein with a *Mw* of 170 kD (P170), which acts as an energy-dependent “drug pump”; it reduces intracellular drug concentration and is associated with multi-drug resistance [163]. *M. charantia* leaf extract was able to reverse the MDR phenotype by increasing the intracellular accumulation of chemotherapeutic drugs [164].

Bitter melon extract induced a significant decrease in the cell viability (>80%) of MDA-MB-231 and MCF-7 cells at concentrations of 2% and 5% while cytotoxicity on primary epithelial cells was negligible; it induces PARP cleavage and caspases activation in MCF-7 cells and the inhibition of apoptotic signaling proteins (survivin, XIAP and claspin) in both cell lines. Moreover, the expression of anti-apoptotic proteins was different, which lead to the conclusion that several signaling pathways are involved in breast cancer cell death [165]. Cell growth of LNCaP was significantly inhibited by *M. charantia* leaf extract through arresting cells in the G1 phase; the extract also inhibited the expression of cyclin D1, PCNA and Bcl-2, and increased cleaved caspase-3 [166]. *M. charantia* can depress cancer cells proliferation in experimental settings; its antitumor activities may be partially attributed to MAP30, α-MMC, β-MMC and other medicinal proteins. In summary, bioactive components of *M. charantia* act as anti-tumor agents mainly through inhibiting tumor cell proliferation, inducing tumor cell apoptosis, influencing energy metabolism, depressing tumor cell metastasis and enhancing the relevant tumor suppressor gene activity (Table 2).

### 3.7. Hypolipidemic Activity

In experimental groups, rats were fed with *M. charantia* at a dose of 140 mg/kg for 30 days, the levels of cholesterol on day 10 were slightly reduced, and a slow reduction was noted in the level of triglycerides after 20 days; supplementation of *M. charantia* significantly decreased the rate of changes in the level of high-density lipoprotein-cholesterol (HDL) and low-density lipoprotein-cholesterol (LDL) (*p* <0.001) [105]. The BMSO (bitter melon seed oil) has been proven to have weight-reducing capability (10 g/kg) in animal experimentation; the indices approached normal control from week 21, and high dose BMSO normalized serum free fatty acid levels in HFD mice after administration; histological changes were also observed as decreased size of adipose cells [169].

Non-esterified cholesterol levels as well as phospholipids were two-times those in the control group in diabetes rats induced by STZ; triglyceride (TG) levels in the experimental group were four-times those in the control group; high-density lipoprotein-cholesterol (HDL-c) levels were 50% of those in the control group. Data evidence suggest that *M. charantia* had an effect in rat serum after 14 days of feeding with 0.5%, 1% and 3% dried powder. Both redced non-esterified cholesterol, cholesterol and TG contents back to normal levels after treatment in a dose-dependent manner. The experiment also found that TG levels in STZ-induced diabetic rat livers and kidneys decreased compared to the normal control group, the phenomenon demonstrated that lipid fluidity was increased or uptake and storage capacity of free fatty acids was decreased in these tissues, thereby causing the serum TG and phospholipid increase [170]. Dietary administration of *M. charantia* consistently elevated HDL-cholesterol levels. Differences in serum lipid parameters (triglyceride, total cholesterol and phospholipid) were demonstrated to be negligible in rats fed the cholesterol-free diet and groups fed with different doses of *M. charantia* unripe fruit powder [170]. After supplemention of *M. charantia* (1.5%), rats in experimental groups showed lower energy efficiency, visceral fat mass, plasma glucose and hepatic triacylglycerol, but higher serum free fatty acids and plasma catecholamines from the fourth week; plasma epinephrine was elevated at seven weeks while steatosis score decreased with a statistically significant difference from the control [171]. For diet-induced obese (DIO) rats, activities of hepatic and soleus muscle mitochondrial carnitine palmitoyl transferase-I (CPT-I) and acyl-CoA dehydrogenase (AD) are elevated; serum adiponectin, uncoupling protein 1 in brown adipose tissue, uncoupling protein 3 in red gastrocnemius muscle and transcription coactivator PGC-1α in both tissues were also significantly elevated after *M. charantia* supplementation [172].

Triterpenoid extracts of *M. charantia* were used for treatment of 3T3-L1 cells. Preadipocyte viability with increasing concentrations (with the exception of concentrations between 0.25 and 0.30 mg/mL) decreased significantly; increasing levels of lactate dehydrogenase indicate that the extract could destroy the integrity of cell membranes. It also led to G2/M block after 48 and 72 h of treatment, and down-regulated PPAR-γ and adiponectin [173].

The potential of *M. charantia* on lowering hepatic triglyceride and cholesterol concentration is mainly attributed to active component(s) in methanolic extracts [174]. The mechanism of steryl glycoside fraction of *M. charantia* inhibits lipid metabolism in vivo lies in the noncompetitive inhibitory effect on corticotropin, glucagon and epinephrine-induced lipolysis by isolated rat adipocytes [53].

### 3.8. Immunomodulatory Activity

*M. charantia* methanolic extracts can significantly promote the secretion of NO and phagocytic activity evaluated via carbon clearance assays in in vivo studies [175]. A water-soluble polysaccharide activated macrophages, splenocytes and thymocytes in vitro, with a maximum effect on NO production and SPI index at a concentration of 200 μg/mL, while the most effective dose to stimulate splenocytes was observed at 25 µg/mL [63].

Studies have shown that after two days of incubation with a dose of 100 µg/mL, α- and β-momordicin have almost no cytotoxic effects on normal cells [41]; the substances have been proven to play an immunomodulatory role by inhibiting the activity of lymphocytes or shifting the kinetic parameters of immune responses [124]; they significantly inhibited mitogenic responses present in mice spleen cells due to the lectin, concanavalin A and the lipopolysaccharides. Momordicin activates and promotes B cell proliferation by inducing surface membrane immunoglobulin activity, while increasing B cell subsets CD86 (cell activation target point) expression, which plays a major role in humoral immunity. In addition, it can induce spleen cells to secrete large amounts of non-specific immunoglobulin IgM after 96 h co-culture and play a role in immune regulation [176]. In vitro, saponins isolated from *M. charantia* may promote IL-2 secretion by varying the ratio of T cells, enhancing phagocytic activity and improving immune function in aging mice [177].

### 3.9. Wound Healing Activity

A series of abnormalities such as impaired immune response and neovascularization, growth factor deficiencies and decreased synthesis of collagen are associated with diabetes and to the delayed wound healing [178]. Treatment with *M. charantia* fruit ointment could significantly enhance wound closure in diabetic rats, and upregulate TGF-β expression in wound tissue, which plays an important role in regulating cell growth and differentiation [179]. For normal experimental animals, methanol extracts also had a similar efficacy and significantly reduced wound area and period of epithelisation [180].

### 3.10. Others

There are also some reports on other bioactivities. Components in *M. charantia* have an inhibitory effect on gastroinstestinal nematodes [181]. Momordin was reported to have hypotensive effects [182]. Administration of ethanolic extracts (500 mg/kg) significantly reduced acetic acid-induced writhing and yeast-induced fever [183]. A fruit extract has been demonstrated to possess activity against *Helicobacter pylori*, which could induce stomach ulcers [184]. Dry powder and volatile oil components of *M. charantia* exhibit strong inhibition on mice skin ulcers induced by alcohol in a dose-dependent manner [185].

## 4. Toxicity and Side Effects

Although the plant is basically harmless to human body under normal conditions, it may induce adverse reactions according to different uptakes, processing methods, physical differences and other conditions. There have been reports of toxicity since 1960s, mainly including acute toxicity, chronic toxicity and reproductive toxicity.

Monthly intake of *M. charantia* leaves was used to prevent childbirth in India [186]. The ethanolic extract of *M. charantia* Linn seed have a greater impact on spermatogenesis and induced histological changes in both testis and accessory reproductive organs of albino mice [187]. For female Wistar rats, aqueous leaf extracts decreased plasma progesterone and estrogen levels in a dose-dependent manner in comparison to the controls [188]. RIPs were also found to have antifertility activity [189]. α-MMC could induce termination of early pregnancy and cause abortion; the probable explanation is the inhibition on development of morulae [190]; β-MMC was also demonstrated to have similar effects, not only influencing embryo adhesion and implantation but also depressing the growth of embryos [191].

Subcutaneous injection of alcoholic extracts mainly induced acute symptoms such as changes in respiratory and heart rates; anatomic results also suggest it led to pathological changes in these organs, and *M. charantia* juice showed a much stronger effect with LC_50_ = 91.9 mg/100 g body weight (b.wt.), compared to alcoholic extracts of 362.34 mg/100 g b.wt [192]. Related research is mainly about the toxicity of subcutaneous injection and reports on oral toxicity are relatively rare. Clinical studies demonstrated that high-dose ingestion (equivalent to 250–500 g) of *M. charantia* fruit caused abdominal pain and diarrhea in diabetes [193]. Moreover, the aqueous extract was reported to significantly decrease hemoglobin concentration of albino rats [194]. *M. charantia* lectin had a cytotoxic effect, which significantly inhibited DNA and protein synthesis in human peripheral blood lymphocytes of normal or leukaemic cells [195]. At the cellular level, 500 and 600 µg/mL TPE was also reported to be toxic to keratinocytes and fibroblasts in vitro [121].

## 5. Conclusions

Up to now, research on the bioactivities of *M. charantia* has developed rapidly. The separation and identification of bioactive components from the plant have attracted more attention, and still maintain an upward trend, while mechanisms in many of the studies still remain to be developed. Clinical studies of the components, especially polysaccharides, should be the focus of research in the long term. With much further research on bitter gourd, the relationship between structure and mechanisms of the efficacy of the various functional constituents will be clarified. At the same time, potential adverse effects should also be investigated further. Firstly, the possible side-effects on the human body, especially long-term consumption, have not been studied. Then, intake of *M. charantia* may also increase the risk of hypoglycemia in diabetic patients. For special populations, taking *M. charantia* should follow the recommendations of doctors or experts. Last but not least, the vast majority of existing studies on bioactive components are performed at the animal and cell levels, hence, their impact on humans has not been demonstrated yet. Therefore, clinical research is needed before their application in relevant industries.

Application of bitter melon in food and pharmaceutical fields are still in the initial processing stages; the health benefits are still far from being fully utilized. Because of its numerous health functions, the plant can be utilized in lowering blood glucose, in tumor therapy and other aspects of clinical applications with broad prospects under the premise of ensuring safety.

## Figures and Tables

**Table 1:** Major bioactive components of *M. charantia* and their related functions.

Major Bioactive Components	Functions	Distribution	Reference
Polysaccharides	Antioxidant, antidiabetic, immune enhancement, neuroprotective, antitumor	Various parts of plants	[31,32,33,34,35,36]
Peptides and proteins	RNA N-glycosidase, polynucleotide adenosine glycosidase (PAG), DNase-like, phospholipase, superoxide dismutase, anti-tumour, immune suppression, antimicrobial	Seed	[37,38,39,40,41,42,43]
Lipids	Antitumor, antioxidant	Seed, flesh	[44,45,46]
Terpenoids	Anticancer, antioxidant, antidiabetic, hypoglycemic, cancer chemoprevention	Stem, leave, fruit	[25,47,48,49]
Saponins	antihyperglycemic, hypolipidmic, antiviral	Fruit, root, seed	[50,51,52,53,54,55,56,57]
Phenolics	Antioxidant, anti-inflammation, immune enhancement	Fruit, pericarp, seed	[58,59,60,61]
Sterols	Antimicrobial	Pericarp, fruit	[15,24,62]

**Table 2:** Suppressing effect on tumor cells of *M. charantia* constituents.

Manifestations	Constituent(s)	Cell Type	Relevant Markers	Mechanisms/Relevant Pathways	Reference
Antiprolifer-ative effect	*M. charantia* seed extract	Su9T01, HUT-102, Jurkat cells	IC50	–	[147]
Induce apoptosis	MAP30	Hep G2	p53, PARP, Bcl2, Bak, JC-1, Bid, caspase-3,8,9	Act through extrinsic and intrinsic caspase pathways	[38,157]
	3β,7β-dihydroxy-25-methoxycucurbita-5,23-diene-19-al (DMC)	(LK) B1-deficient MDA-MB-231	Cyclin D1, CDK6, Bcl-2, XIAP, cyclooxygenase-2, NF-κB	PPARγ-targeted signaling pathways	
Influence energy metabolism	Bitter melon juice	BxPC-3, MiaPaCa-2, AsPC-1, Capan-2	Caspases, Bcl-2, cytochrome c, survivin, p21, phosphorylated MAPKs	MAPK pathway	[153,154,167]
	Methanol extract of *M. charantia* (MCME)	Hone-1, AGS, HCT-116, CL1-0	caspase-3, DFF45, PARP, Bax, Bcl-2	Caspase- and mitochondria-dependent pathways	
	Bitter melon extract (BME)	PC3, LNCaP	Cyclin D1, cyclin E, p21, Bax	MEK–ERK and p38 MAPK pathway	
Depress tumor cell metastasis	Kuguacin J	PC3	MMP-2, MMP-9, uPA	Inhibition of the expression of Akt, β-catenin, and MMPs	[151,152,168]
	*M. charantia* leaf extracts (BMLE)	PLS10			
	MCME	CL1-0, CL1-5	MMP-2, MMP-9, Src, FAK		
Reverse MDR	*M. charantia* leaf extracts	KB-V1	Resistance to vinblastine	Inhibition of P-glycoprotein activity	[164]

## Abbreviations

DMBA	Dimethybenzanthracene
uPA	Urokinase type plasminogen activator
iNOS	Inducible nitric oxide synthase
AMPK	Adenosine 5′-monophosphate (AMP)-activated protein kinase
TPA	12-*O*-tetradecanoyl-phorbol-13-acetate
PARP	Poly ADP-ribose polymerase
XIAP	X-linked inhibitor of apoptosis protein
MAPK	Mitogen-activated protein kinase
NF-κB	Nuclear factor kappa-light-chain-enhancer of activated B
HX-XO	Hypoxanthine-xanthine oxidase
PPAR	Peroxisome proliferators-activated receptors
NO	Nitric oxide
SPI	Splenocyte proliferation index
ABTS	2,2′-azino-bis(3-ethylbenzothiazoline-6-sulfonic acid)
Mw	Molecular weight
HDL	High-density lipoprotein-cholesterol
LDL	Low-density lipoprotein-cholesterol
DPPH	1,1-diphenyl-2-trinitrobenzene hydrazine
AST	Aspartate aminotransferase
ALT	Alanine aminotransferase
SOD	Superoxide dismutase
CAT	Catalase
GST	Glutathione S-transferase
